# Promising on-site and rapid SARS-CoV-2 detection *via* antigens

**DOI:** 10.3389/fpubh.2022.978064

**Published:** 2022-10-10

**Authors:** Jian Zhang, Haochen Qi, Jayne Wu, Xiaochun Guan, Zhiwen Hu, Lei Zheng

**Affiliations:** ^1^College of Electrical and Electronic Engineering, Wenzhou University, Wenzhou, China; ^2^School of Food and Biological Engineering, Hefei University of Technology, Hefei, China; ^3^Department of Electrical Engineering and Computer Science, The University of Tennessee, Knoxville, TN, United States; ^4^School of Computer and Information Engineering, Zhejiang Gongshang University, Hangzhou, China

**Keywords:** SARS-CoV-2 detection, antigen test, point-of-care diagnosis, on-site detection, spike protein, nucleocapsid protein

## Challenges and strategies for SARS-CoV-2 diagnosis

COVID-19 pandemic over the past years has shown a great need for rapid, low-cost and on-site detection of severe acute respiratory syndrome coronavirus 2 (SARS-CoV-2). At present, polymerase chain reaction (PCR) based nucleic acid test (NAT) has been a gold standard in clinical practice (1), which has high sensitivity and high throughput. It is capable of providing quantitative results (with qRT-PCR), and recognizing viral mutations with short turnaround (2–4). While NAT plays an irreplaceable role in epidemic prevention around the world, the associated high equipment-cost, the need to operate in a laboratory setting and the longer than desired turnaround time are driving the research effort to achieve low-cost and point-of-care testing of viral infections.

Besides nucleic acids, antibodies and antigens are the two other types of targets for SARS-CoV-2 diagnosis (1). Antibodies such as IgM and IgG are often evaluated using established assays as an auxiliary to NATs, but the appearance of antibodies in the body is a lagging indicator for infection and does not always correlate with the presence of viruses (5). Therefore, antibody tests alone are not reliable for accurate detection of viruses, particularly in the early stage of infection before antibody appearance. Clinically, they have been used for immune evaluation after vaccination and postmortem analysis in asymptomatic individuals (6). To increase the reliability of detection, NAT can be combined with antibody tests to cover a wider range of disease progression. This approach has been employed to detect a variant of SARS-CoV-2 (7), which in fact illustrates that antibody cannot act as an independent indicator for virus presence.

**Figure 1:**
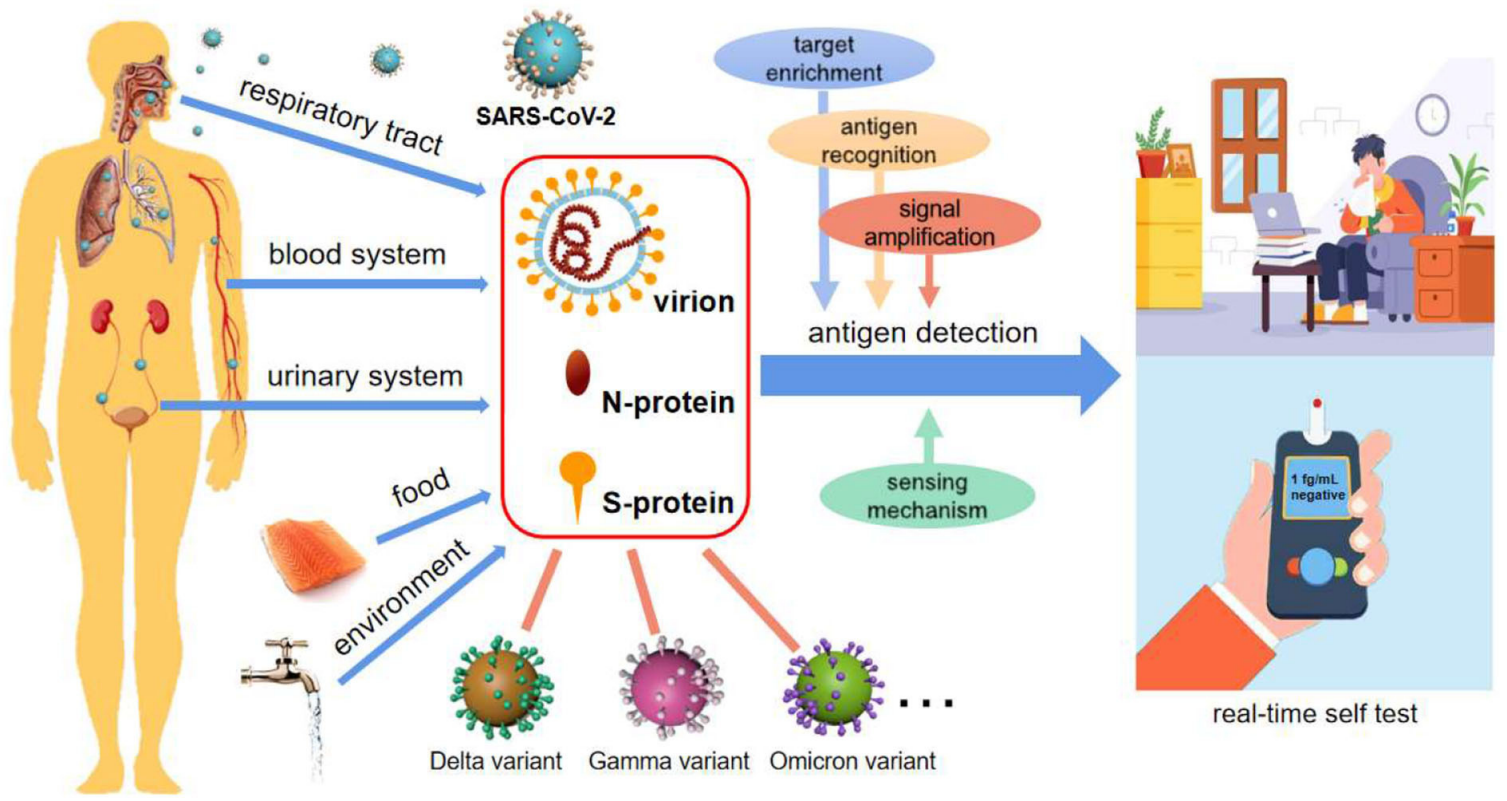
SARS-CoV-2 diagnosis *via* antigen test. Antigen detection toward POCTs commonly uses virion, N-protein, and S-protein as targets, potentially integrating with target enrichment, antigen recognition, signal amplification as well as innovative sensing mechanisms. Antigen-based detection strategies may be applicable for various types of body fluids, food, and environmental samples, and are broad-spectrum for SARS-CoV-2 variant recognition. It is also an enabler of real-time self-tests for infectious diseases.

To make the diagnosis more affordable, more convenient and more rapid, antigens from viruses have been attracting attention as biomarkers for SARS-CoV-2 identification (8–10). Generally, there are three common antigens for SARS-CoV-2 detection, i.e., live virion (8), spike (S-) (9) and nucleocapsid (N-) proteins (10) (Figure 1). The latter two are both important structural proteins of the virion. S-protein on the virion surface is responsible for binding to the host cell receptor and fusing the membranes of virus and cell (11), and N-protein is an abundant RNA-binding protein for viral genome assembly and release (12). Focusing on emerging diagnostic tools, this opinion highlights and summarizes the recent advances in antigen detection techniques, in hope of providing a strategic reference for real-time and high throughput detection of SARS-CoV-2.

## Representative emerging techniques for antigen detection

Few methods have been reported to directly detect live virions of SARS-CoV-2 until a unique aptasensor is developed (8), which can accurately recognize the surface of live virions from the altered surface of inactivated virions. It reaches a limit of detection (LOD) of 10^4^ copies/ml, and the test time is from 30 min to 2 h. This detection is easy to operate with a simple protocol since sample pretreatment is not required. In comparison, some biosensors have been reported with S-protein as the target, such as the one employing human angiotensin-converting enzyme 2 (ACE2) as a probe (9), which successfully recognizes the SARS-CoV-2 UK variant 1.1.7. B. It yields a result within 6.5 min, meeting the requirement of on-site diagnosis, and the cost of this biosensor is only 1.5 US dollars. Another impressive immunosensor is for food quarantine (13), with an ultralow LOD of 10^−6^ ng/ml acquired in 20 s. The cost of this sensor is about 1 US dollar. The rapid response and low cost make it possible to realize large-scale and real-time virus screening in food and environmental media.

Due to the long-term preservation of N-proteins in the body, more emerging strategies are based on N-protein detection. One representative technique is a mass spectrometry-based system (14). Although this platform is relatively complex and expensive, it allows multiplexed analysis of four samples within 10 min, enabling the processing of more than 500 samples per day. This method has also been qualitatively and quantitatively validated using 985 specimens previously analyzed by real-time RT-PCR, with an accuracy of 84% and a specificity of 97%. Another typical immunosensor is based on magnetic nanobeads (15), which achieves a LOD of pg/ml level in serum within 1 h. In this research, a smartphone-based diagnostic system has also been developed for point-of-care tests (POCTs). Recently, an aptasensor for ultrasensitive N-protein detection is reported (16) achieving an ultralow LOD of 10^−6^ ng/ml, a response time of 15 s, and a cost below 1 US dollar. The matrices include water, saliva, even serum and plasma. This sensor is competitive for low-cost screening and POCT applications.

## Merits of antigen detection

Herein, some prospects of antigen detection strategies are explored based on limited investigation. It is worth noting that ingenious approaches for antigen-based SARS-CoV-2 detection have been extensively researched in the last 2 years. With immunoreactions and key-lock space conformations, the process of recognizing antigens by antibodies, aptamers or enzymes is very fast owing to the binding in several seconds between probes and targets. As a result, antigen detection is rapid, and can even be shortened to dozens of seconds (13, 16). Small, inexpensive, and simple-to-use sensors, as well as innovative sensing mechanisms, make compact platforms possible (9, 13, 16) (Figure 1). Among the antigens, widely adopted N- and S-proteins are of high abundance in not only the respiratory tract, but also blood and other body fluids, and even external matrices (Figure 1), supporting more medium types and analytical methods (8, 9, 16). As we have seen, these studies are forming a hotspot due to the strengths of antigen detection in realizing POCT and large-scale environmental screening of SARS-CoV-2.

Another issue is the quantification of viral load, which is essential to evaluating disease course and infectivity. The samples for SARS-CoV-2 detection are typically collected by swabs, which does not correspond to a well-defined sampling volume. According to existing reports, the viral RNA can hardly be detected in blood in most infected patients (six of 57 patients) (17), while antigen is always found in blood or other body fluids (10). Using suitable protocols, antigen-based detection has been reported to be quantitative and of a wide linear range covering at least three orders of magnitude (8, 9, 13–16).

Over just 2 years, many SARS-CoV-2 mutations, such as Delta and Omicron, have appeared around the world, as expected for an RNA virus, which poses new challenges to their detection, particularly at the beginning of a mutation emergence. In contrast to accurate recognition by RNA sequencing, antigen detection cannot easily distinguish congeneric proteins from different variants. On the other hand, the structural proteins are usually well-preserved, particularly for N-protein (12, 16). Based on this, antigen-testing strategies are capable of broad-spectrum recognition of these variants (Figure 1), which has been verified by many clinical sample tests (9, 18). Due to the lack of specific medicine for particular variants, identifying which mutated strains cause the spread of Covid is of limited importance. In this context, broad-spectrum detection of the virus will be an efficient strategy to screen and identify the infectors.

Similar to all the bioassays, the key figure of merit for SARS-CoV-2 detection should be the accuracy. Presently, a common perception is that the accuracy of antigen tests is low despite these tests being rapid (1). However, this view may not be supported by solid evidence. The accuracy of antigen detection depends on the specific and reliable binding between probe and biomarker, and specific probes can be identified through established antibody and aptamer screening methods. As a result, the reported accuracy of COVID-19 diagnosis using antigen-positive samples has been demonstrated to be good (84%−100%) (9, 14, 15).

As a matter of fact, a variety of techniques have been implemented for antigen-based detection, which invariably leads to a large variation in sensitivity. Many research groups are working diligently to enhance the detection sensitivity. Advanced techniques, such as microfluidics, have successfully demonstrated enrichment of ultra-trace antigens in unprecedented ways, achieving ultralow LODs of 10^−6^ ng/ml for S- and N-proteins (13, 16), i.e., the concentrations of 10^4^ molecules/mL. Assuming that every virion corresponds to 20 protein molecules, the virion concentration is roughly estimated to be 500 #/ml, which is on par with PCR based NATs. Other technological advances, including innovative sensing mechanisms and signal amplifications (13, 15, 16), also contribute to improved sensitivity of sensors and assays.

To concisely and clearly illustrate the main features of antigen tests for SARS-CoV-2 detection, Table 1 presents a summary of the targets, specimens, turnaround time, LOD, time window for test, and brief comments.

**Table 1:** Features of antigen tests for SARS-CoV-2 detection.

**SARS-CoV-2 detection strategy**	**Targets**	**Specimens**	**Turnaround time**	**LOD**	**Time window for test**	**Comments**
Antigen test	Live virion, N-protein, and S-protein	Upper respiratory secretion, saliva, serum, plasma, other body fluids, water, food	Minutes to hours	fg/ml to ng/ml	Whole disease course	• Easier to implement outside laboratory with portable sensors and apparatuses • Rapid and low-cost assays enabling POCT and large-scale screening • Capable for virus presence test in various matrices • Broad-spectrum response to variants • Lack of direct correlation with viron load • Weak ability to identify specific variants • Not enough products for clinical applications

## Summary and prospect

Some prospects of antigen detection strategies have been explored based on the limited investigation. First of all, the advantages and importance of NATs are undeniable, which is supported by world-wide adoption of NATs as the primary detection tool of COVID-19. At the same time, considerable efforts have been dedicated to the research and development of a variety of improved or new detection approaches. Only by balancing the advantages and disadvantages of various detection assays according to specific purposes can we obtain the most economical and optimal option. Antigen tests are becoming a promising strategy owing to their merits of rapid response, low cost and simple operation. With the integration of emerging technologies, antigen tests are conducive to be implemented as POCTs on a large scale and as a strong auxiliary test to NATs. Of course, there are still limitations and shortcomings with antigen based techniques at the present stage. One is the large variation in sensitivity from various research groups and developers. The most sensitive technique reports comparable sensitivity to NATs, while the majority of techniques have much higher LODs. Another one is a lack of standard for viral load calibration compared with NATs, which is due to the insufficient products for clinical applications.

Currently, antigen detection technology is undergoing a period of rapid development. Although they have not been popularized around the world, antigen tests hold great promise for low-cost and on-site SARS-CoV-2 screening and diagnosis, particularly in communities and regions with limited resources. We anticipate revolutionary breakthroughs in both academic and clinical fields in near future. In practical applications, multiple methods are often combined to minimize the drawbacks of a single method, and antigen tests can be expected to act as a preliminary screening tool, even by self-test, prior to NATs results. With the rapid development of new technologies and methods, we believe that more sensitive, efficient and mature antigen detection methods and products will appear in the near future, which would provide powerful tools for public health.

## Author contributions

JZ and JW wrote the original draft. JW, HQ, and LZ revised the draft. All authors contributed to the article and approved the submitted version.

## Funding

This work was funded by the National Natural Science Foundation of China (62074047 and 32072306) and USDA NIFA (The United States Department of Agriculture, the National Institute of Food and Agriculture) (Grant No. 2017-67007-26150).

## Conflict of interest

The authors declare that the research was conducted in the absence of any commercial or financial relationships that could be construed as a potential conflict of interest.

## Publisher's note

All claims expressed in this article are solely those of the authors and do not necessarily represent those of their affiliated organizations, or those of the publisher, the editors and the reviewers. Any product that may be evaluated in this article, or claim that may be made by its manufacturer, is not guaranteed or endorsed by the publisher.
